# Model Lightweighting for Real-time Distraction Detection on Resource-Limited Devices

**DOI:** 10.1155/2022/7360170

**Published:** 2022-12-23

**Authors:** Jing Wang, ZhongCheng Wu

**Affiliations:** ^1^High Magnetic Field Laboratory, Hefei Institutes of Physical Science, Chinese Academy of Sciences, Hefei 230031, China; ^2^University of Science and Technology of China, Hefei 230026, China

## Abstract

Detecting distracted driving accurately and quickly with limited resources is an essential yet underexplored problem. Most of the existing works ignore the resource-limited reality. In this work, we aim to achieve accurate and fast distracted driver detection in the context of embedded devices where only limited memory and computing resources are available. Specifically, we propose a novel convolutional neural network (CNN) light-weighting method via adjusting block layers and shrinking network channels without compromising the model's accuracy. Finally, the model is deployed on multiple devices with real-time detection of driving behaviour. The experimental results for the American University in Cairo (AUC) and StateFarm datasets demonstrate the effectiveness of the proposed method. For instance, for the AUC dataset, the proposed MobileNetV2-tiny model achieves 1.63% higher accuracy with just 78% of the model parameters of the original MobileNetV2 model. The inference speed of the proposed MobileNetV2-tiny model on resource-limited devices is on average 1.5 times that of the original MobileNetV2 model, which can meet real-time requirements.

## 1. Introduction

According to the Association for the World Health Organization (WHO), more than 1.35 million people die in road traffic accidents every year [1]. Distracted driving has been one of the leading causes [2]. The National Highway Traffic Safety Administration (NHTSA) reports that 3,142 people were killed on US roads involving distracted drivers in 2019 [3]. It is necessary to design a system to detect distractions, helping alleviate the current serious situation.

A visual feature-based approach to capturing distraction behaviour has been widely used in intelligent transportation systems with the help of deep neural networks. At present, edge-based advanced driver assistance (ADAS) [4] or driver status monitoring (DSM) [5] systems are now an important module for collaborative driving. The edge central processing units (CPUs) and graphics processing units (GPUs) of systems are generally powerless [6]. Ziran et al. [7] proposed a vehicle-to-cloud method for driver assistance systems. Abdu et al. [6] deployed the training and validation modules in the cloud environment; that is, the edge device data are directly uploaded to cloud and the distracted driver detection task is performed on cloud servers, where abundant computing and storage resources are available to realize real-time inference [8].

However, several flaws in cloud computing make it less favourable for applications enabled by edge devices. First, the speed of data transmission highly depends on the Internet connection. Especially for some high-speed moving scenes, for example, in vehicles, data transmission may not be complete in the case of poor network signals. More importantly, as the number of edge devices is growing exponentially, data transmission speed is becoming the bottleneck of cloud computing. Second, high-end GPUs are expensive, bulky, and have high power consumption, which makes it challenging to deploy devices on resource-limited edge devices. Third, data transmission to a centralized cloud server also leads to security and privacy issues [9]. Hence, it is necessary to process the data at the site where it is collected rather than in “cloud computing” [10].

One of the current research studies on detecting distracted driving on edge devices with limited resources is the pruning method that introduces filters and weights on large CNNs such as ResNet50 [11] and VGG [12]. However, the large pruning ratio easily leads to a loss of accuracy. Another suitable approach is introducing lightweight models such as MobileNetV2 [13]. For example, MobileNetV2 is seven times faster than ResNet50 but has a 3.6% lower accuracy. However, due to the low hardware utilization of compact operators commonly adopted by these lightweight models, existing lightweight models are still limited in their ability to improve practical hardware efficiency.

This paper focuses on detecting distracted driving with resource-limited edge devices. We design a new strategy to develop efficient CNNs that maintain model accuracy while increasing inference speed. Specifically, the blocks and channels of the network are optimized by analyzing the sensitivity of the model's performance. The innovation of our method is compatible layer pruning and filter pruning to obtain a novel model lightweight way. The experiments showed that our approach can be applied to in-vehicle terminals to provide real-time reminders.

The contributions are summarized as follows:

1. Block-level architecture redesign is proposed to make the model more suitable for driving behaviour analysis scenarios. Unlike the direct compression of the block layer in the past, the distribution of the block layer is automatically adjusted according to the task, which helps the network learn more abstract and detailed features to improve its accuracy. Sensitivity information is used to adjust the number of residual bottleneck block layers.
2. Channel-level architecture redesign is proposed via pruning filters at each layer of the network with dynamic pruning ratios. Those filters of “relatively little” importance are pruned to compress the CNN model. Fine-tuning is applied to reduce the loss of accuracy.
3. We conduct extensive experiments on multiple lightweight CNNs on the AUC and StateFarm-distracted driving datasets, and our method achieves accurate, fast, and lightweight CNNs. At the same time, our model is deployed on resource-limited devices, which confirms that our method can achieve the effect of real-time distraction detection processing.

For the AUC dataset, the proposed method applied to the MobileNetV2 network achieves 1.63% higher accuracy than the original MobileNetV2 network with only 2.78 M parameters. The results show 99.81% accuracy for the StateFarm dataset. The detection speeds of the Xiaoyi Smart Rearview Mirror equipment and HUAWEI MediaPad c5 device are almost 1.5 times faster than before.

## 2. Related Work

### 2.1. Distracted Driver Detection

Due to the increasing number of traffic accidents caused by distracted driving, detecting distracted driving has attracted considerable attention from the research and the industry community. Zhao et al. [14] created the Southeast University Driving Posture (SEU-DP) dataset in 2011, which includes four types of behaviours: safe driving, operating the shift lever, calling and eating, and talking on a phone. K-nearest neighbour (KNN), random forest (RF) [15], Geronimo–Hardin–Massopust (GHM) multiwavelet transform [16], and the pyramid histogram of oriented gradient (PHOG) [17] methods were used for driving posture feature extraction and distraction detection. However, the SEU-DP dataset was not publicly available.

The StateFarm dataset was the first published dataset in the distracted driving detection competition on Kaggle in 2016, which contains ten types of distracted driving behaviours: driving safely, texting with the right hand, calling with the right hand, texting with the left hand, calling with the left hand, operating the radio, drinking, reaching back, hair and makeup, and talking with passengers [18]. Abouelnaga et al. [19, 20] created the AUC-distracted driver dataset, which contains the same ten types as the StateFarm dataset, in 2019. Compared to traditional machine learning methods such as RF and KNN, the CNN method can effectively improve accuracy and handle more complex classification problems. More and more researchers tend to use deep learning to solve this problem. The visual geometry group (VGG) [21], InceptionV3 [22], ResNet [23], and video-based methods [24] were used to improve distraction detection accuracy. Improved ReSVM [25] is a method that combines in-depth features from ResNet with a support vector machine (SVM) classifier for driver distraction detection.

To improve recognition accuracy, facial expressions, hand gestures [26], and human body key points [27] are also used in the feature extraction of driving behaviour. Chiou et al. [28] used a cascaded face detector to detect the face area and obtain the coordinates of the face, eyes, and mouth to judge whether the driver was driving normally or not according to the coordinates. When abnormal driving behaviour is detected, it is further determined whether the behaviour is drowsy driving behaviour or distracted driving behaviour. Facial landmark detection [29] was used for distraction detection due to the driver's head panning. The proposed method increases the feature extraction capability through novel geometric and spatial scale-invariant features and outperforms the existing state-of-the-art approaches in detection accuracy for multiple datasets.

However, early distraction detection research focused on improving recognition accuracy. Most current studies are based on traditional deep CNN models [30], such as ResNet and VGG. Although they can achieve high accuracy, they are not friendly to embedded systems with limited memory space and computing resources. At present, some researchers have begun to study distraction detection lightweight methods. Binbin et al. [31] proposed a new neural network model based on decreasing the size of the filter. The model had only 0.76 parameters, and the results were 95.59% and 99.87% accurate for the AUC and StateFarm datasets, respectively. Dropout, L2 regularization, and batch normalization [32] were used on VGG-16 to reduce the number of parameters from 140 M to 15 M only. Bhakti et al. [33] proposed a new structure network mobileVGG with only 2.2 M parameters. Zuopeng et al. [34] introduced a lightweight microscopic detection network (LMS-DN) for lightweight distraction detection.

At present, the research on the lightweight of distracted driving is relatively scarce and constantly developing. Therefore, we were committed to finding a novel lightweight method for resource-limited devices to detect distracted driving in real time. This paper focuses on reducing the number of parameters and increasing speed while maintaining high accuracy.

### 2.2. CNN Compression Techniques

To deploy CNNs on resource-limited devices, pruning [35] has often been used to reduce the model's complexity while trying to maintain decent accuracy.

Many model pruning methods for filters and weights have been proposed in recent years [36]. Hao et al. [35] proposed reducing the number of convolutional channels in the convolutional layer to reduce the size of the model and computational complexity. The L1-norm statistic was used to select less significant filters. Sensitivity information was used to evaluate the influence of each layer of the network. The FPGM strategy [37] was proposed to assess the importance of filters in a single convolution by calculating the geometric distance between filters. Liu et al. [38] suggested that researchers consider model pruning as a model design process, using different pruning rates on different layers according to various tasks. Learning filter pruning criteria [39] were proposed to learn and select the most appropriate pruning criteria for each functional layer.

Compared with filters and weights pruning, pruning an entire layer/block is more effective in reducing model complexity and hardware latency [40]. Block-level pruning [41] adopts a multiobjective optimization paradigm to reduce the blocks of the model while avoiding accuracy degradation. Xu et al. [42] proposed a fusible residual convolutional block for layer pruning. The convolutional layers of the network were converted into a residual convolutional block with a layer scaling factor for layer pruning. A DepthShrinker framework [43] was proposed by shrinking the basic building blocks of CNNs with irregular computation patterns into dense networks.

However, the previous work was mainly based on pruning large models. Compared with the pruning of large models, pruning of lightweight networks such as MobileNetV2 is more difficult. In this paper, we propose a new lightweight model compression method for MobilevetV2. This method will be compatible with block-level pruning and channel-level pruning. Different from previous block-level pruning methods, we mainly prune the multigroup residual modules for networks.

### 2.3. Resource-Limited Device

Resource-limited devices, such as embedded devices, mobile devices, and other Internet of things devices [44], have limited memory and processing resources. Deep learning algorithms are often computationally and memory-intensive and, therefore, unsuitable for resource-limited devices [45]. Computational processing units on resource-limited devices typically include integrated CPUs [46] and GPUs [47]. Extensive research is underway to develop suitable hardware acceleration units, such as FPGA [48], ASIC [49], TPU [50], and NPU [51], to create distributed systems to meet the high computational demands of deep learning models.

Another solution is to use lightweight networks, such as MobileNetV2, SqueezeNet [52], MobileNetV3 [53], and EfficientNet [54], which enable feasible embedded deployment. However, these lightweight models are still difficult to balance in terms of speed and accuracy. MobileNetV2 is 3.6% less accurate than ResNet50.

In this work, we designed an improved method based on the lightweight model that considers the speed and accuracy of inference. We chose two devices: the Xiaoyi Smart Rearview Mirror equipment and HUAWEI MediaPad c5 device, which can be used for the actual car driving recorder intelligent system. With the resource-limited CPU and GPU, the devices can be used to verify the effectiveness of our method in edge device deployment.

## 3. Method

**Figure 1:**
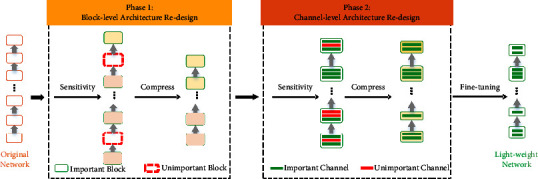
An overview of our framework.

The overall framework of the proposed model is shown in Figure 1. First, L1-norm regularization and sensitivity methods are used for block-level architecture redesign to improve accuracy. Then, filter pruning and fine-tuning methods are introduced for channel-level architecture redesign to reduce the number of parameters. The model is optimized and trained on the server side. The improved method is deployed on embedded systems for model inference. We use MobilevetV2 as the basic model for the experiment and, at the same time, we apply our improved method to multiple lightweight models, such as SqueezeNet [52], MobileNetV3 [53], and EfficientNet [54].

### 3.1. L1-Norm and Sensitivity

L1-norm regularization was used to select pruned filters. Let *X*_*i*_ ∈ *ℝ*^*n*_*i*_×*w*_*i*_×*h*_*i*_^ denote the input feature maps for the *i* th convolutional layer and *X*_*i*+1_ ∈ *ℝ*^*n*_*i*+1_×*w*_*i*+1_×*h*_*i*+1_^ be the feature maps for the next convolutional layer. *n*_*i*+1_ 3D filters *F*_*i*,*j*_ ∈ *ℝ*^*n*_*i*_×*k*×*k*^ are applied to the *i* th convolution layer. *k* × *k* is the size of the convolution filter.

The L1-norm of *F*_*i*,*j*_ is

(1) $$\begin{array}{c} F_{l}=||F_{i,j}{||}_{1}\text{.} \end{array}$$

The relative importance of the filter *F*_*i*,*j*_ can be measured by calculating the sum of its absolute weights, and the sum of *F*_*i*,*j*_ absolute weights is

(2) $$\begin{array}{c} s_{j}=\sum \left|Fi,j\right|\text{.} \end{array}$$

Based on L1-norm regularization, the sum of the absolute weights of *F*_*i*,*j*_ shows the weight to the magnitude of the output feature map. Compared with other filters in this layer, filters with smaller absolute weights tend to generate feature maps with weak activation. The smaller the absolute weight result, the less significant the filter.

By pruning each layer independently, each layer's sensitivity can be understood. The sensitivity of each layer of the convolutional network could be used to represent the importance of the convolutional network layer, which can visually display the impact of each layer of the network and filter on accuracy. Sensitivity is obtained by pruning the filters of each layer independently and evaluating the accuracy of the verification set. After calculating the sensitivity, less essential layers/filters will be pruned first.

### 3.2. Block-Level Architecture Redesign

#### 3.2.1. Motivation

Sensitivity was analyzed to understand the applicability of MobilenetV2 in distracted detection tasks. MobileNetV2 contains 19 residual bottleneck blocks, of which multiple residual bottleneck blocks are generated in a cycle. Taking the residual unit of MobilenetV2 as a whole bottleneck block, the method only counts the sensitivity of the first-layer network of the residual unit. The sensitivity of the AUC dataset can be obtained by applying different pruning ratios on the convolutional layer.

Sensitivity analysis can be used to optimize existing open-source models for driving behaviour analysis scenarios. We observe that the sensitivity of layers in the same cycle stage (with the same feature map size) is different. The more the number of cycles, the smaller the contribution of the subsequent layers to accuracy. Layers with relatively flat slopes are less sensitive for accuracy. We can reduce the model sublayers that have a relatively small impact on accuracy. At the same time, for precision-sensitive layers, we can increase the number of cycles of the module sublayer to improve precision. Our idea is conducive to improving the accuracy of MobileNetV2 in driving behaviour tasks, given that the distribution of MobileNetV2's block layers is designed based on the benchmark dataset.

#### 3.2.2. Block-Level Optimization

Our method optimizes the layout of the network layer from a well-trained model to improve accuracy and increase the model's applicability in specific task scenarios. For the network structure of MobilenetV2, it is mainly to optimize the number of cycles of blocks in the same stage.

For multiple cycles of residual blocks, the number of cycles can be adjusted according to the influence of the sensitivity results on accuracy. For the residual block with a slight change in sensitivity, the number of cycles can be reduced,the residual block of the cycles whose impact on the accuracy is controlled within k%. For the residual block with a significant change in sensitivity, the number of cycles can be increased until the increased cycle modules have less than k% impact on accuracy.

Nevertheless, layer-by-layer optimization and retraining can be a very time-consuming process. We design an automatic optimization adjustment strategy, which can calculate the importance score of each layer using the same sensitivity analysis criteria and adjust the distribution number of block layers of the task adaptively according to the value of *k*. Our strategy achieves comparable accuracy to the original.

At the same time, a fine-tuning process is introduced to speed up the training process of the tuned network. Same as the original training process parameters, the fine-tuning process is obtained by retraining on the basis of original network weights. By adopting block-level optimization, the network can increase its adaptability to specific tasks.

**Figure 2:**
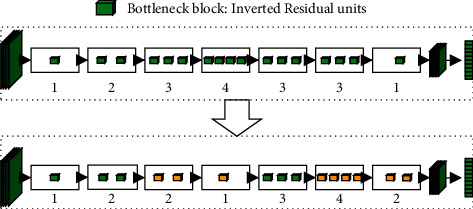
The block-level optimization of MobilenetV2 network layers.

Figure 2 shows the optimization of the layout of MobilenetV2 network layers. The layers of con4_3, con5_2, con5_3, and con5_4, which have a relatively small impact on accuracy, were reduced. The residual unit after con7_3 and con8, which significantly affected accuracy, was increased. The adjustment of the block layer, which we designed, can automatically adjust the distribution number of the block layer according to the task. In particular, increasing the number of deep block layer loops helps the network learn more abstract and detailed features to improve accuracy.

### 3.3. Channel-Level Architecture Redesign

The sensitivity analysis helps explain the principle of network layer optimization and improve the model's accuracy. However, many network filters after network layer optimization still do not contribute much to the final result and appear redundant. The method of pruning filters is used to compress the network.

In our algorithm, the pruning ratio is dynamically calculated based on sensitivity information, and the pruning ratio of each layer filter is different. The dynamic pruning rate ensures that the accuracy loss after pruning is as tiny as possible. According to the sensitivity information, the impact of the pruning ratio of each layer filter on accuracy is controlled within k%. Fine-tuning is used after pruning to ensure that the accuracy loss of the network after pruning is as tiny as possible.

The procedure of pruning is as follows:

  Step 1: we calculate the pruning ratios of each convolutional layer filter based on sensitivity information.
  Step 2: We set different pruning ratios for each convolutional layer filter. We prune a small number of filters layer by layer and count float point operations (FLOPs) and accuracy. If FLOPS and accuracy have met the requirements, we go to Step 4. Otherwise, we go to Step 3.
  Step 3: we perform epoch fine-tuning on the network and enter Step 2.
  Step 4: we perform fine-tune training to convergence.

### 3.4. Overall Optimization

We get the final distribution for all layers with block-level and channel-level optimization. The pruned network is then fine-tuned to obtain the final accuracy. The whole process of the proposed CNN light-weighting method is explained in Algorithm 1.

## 4. Results and Discussion

The experiments use the AUC dataset and the StateFarm dataset to verify the effectiveness of the improved method. Finally, the model is deployed on embedded devices for validity and real-time testing. This improved design could achieve real-time goals with lower computational complexity and memory requirements while maintaining good driver posture classification accuracy. The top-1 accuracy, the approximate number of parameters, FLOPs, and frames per second (FPS) were used to evaluate the performance of the proposed design.

### 4.1. Datasets

**Figure 3:**
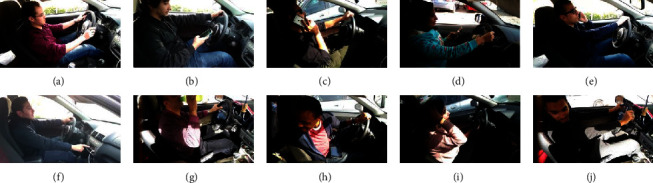
Sample images of ten types of distracted driving behaviours in the AUC dataset. (a) Driver safe. (b) Text right. (c) Talk right. (d) Text left. (e) Talk left. (f) Adjust the radio. (g) Drink. (h) Reaching behind. (i) Hair and makeup. (j) Talk to passengers.

The AUC dataset contains ten types of distracted driving behaviours.  Figure 3 shows the sample images of ten types of distracted driving behaviours from the AUC dataset. The camera was placed on the upper right of the front passenger seat to record the simulated behaviour of the driver. The videos were cut into single-frame pictures, with each frame size of 1080 × 1920 pixels. The dataset has 31 participants from 7 different countries, with 17,308 images, including 12,977 images in the training set and 4331 images in the test set.

**Figure 4:**
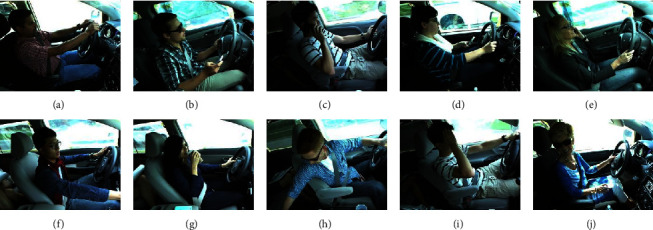
Sample images of ten types of distracted driving in the StateFarm dataset. (a) Driver safe. (b) Text right. (c) Talk right. (d) Text left. (e) Talk left. (f) Adjust the radio. (g) Drink. (h) Reaching behind. (i) Hair and makeup. (j) Talk to passengers.

The StateFarm-distracted driving detection dataset was used in the experiment to verify the method's applicability. The StateFarm dataset has 26 participants (13 males and 13 females), including 22,424 training set images and 79,726 test set images, and each image has a size of 640 × 480 pixels.  Figure 4 shows the sample images of ten types of distracted driving behaviours from the StateFarm dataset. After the competition, the labels of the test set were not available. The training set was processed in two groups of experiments: In the first set of experiments, the StateFarm training set was randomly divided into a 90% training set (17934 images) and 10% validation set (4490 images) for performance evaluation and verification. In the second set of experiments, the StateFarm training set was divided into 70% and 30% randomly referring to other literature methods. In the third set of experiments, the StateFarm training set was randomly divided into a 60% training set, 20% test set, and 20% validation set for the cross-validation experiment.

### 4.2. Results for the AUC and StateFarm Dataset

The method was developed in PaddlePaddle [55] with the programming language Python3.7. A single NVIDIA GeForce GTX 1080Ti GPU with 12 GB system RAM was used to train the network. The cosine function was adopted as the function of learning rate decay in the training process. The learning rate was 0.05, the epoch was 100, and the image shape was [3, 224, 224]. The batch size was set to 32. The optimizer used SGD utilized with momentum = 0.9 and weight decay = 5 × 10^−3^. The ImageNet pretrained model was used as initialization to speed up model convergence.

Table 1 shows the change in the residual bottleneck block of MobileNetV2 from *n* to *n*′ in the global block layout optimization step, with channels changing from *c* to *c*′ in the pruning step. Each line describes a sequence of 1 or more residual bottleneck layers, repeated from *n* times to *n*′ times. *n*′ is the number of cycles adjusted according to the sensitivity result. This optimization can improve the accuracy of the network in detecting distracted driving. *c*′ is the channel of each block improved by the pruning step. The feature maps for each convolutional layer can drop at least 10% of channels without affecting accuracy. Reducing the number of filters can effectively reduce the number of parameters.

**Figure 5:**
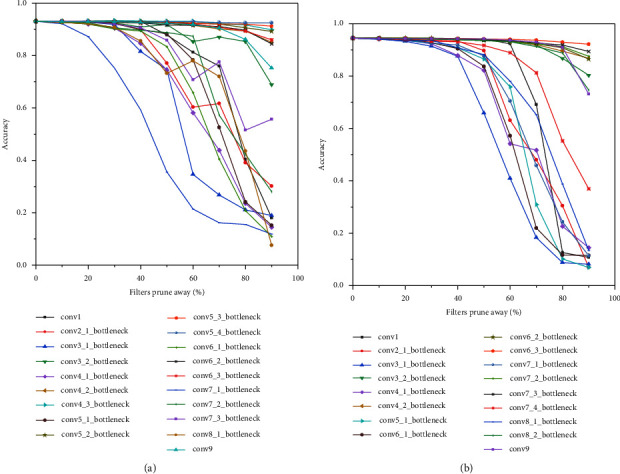
The sensitivity of MobilenetV2 and MobilenetV2-tiny for the AUC dataset. (a) The sensitivity of MobilenetV2. (b) The sensitivity of MobilenetV2-tiny.

Figure 5 shows the sensitivity of MobilenetV2 and MobilenetV2-tiny for the AUC dataset. The abscissa is the ratio of filters cropped, and the vertical coordinate is the loss of accuracy. Each coloured dotted line represents a convolutional layer in the network. It shows the relationship between the accuracy and the pruning ratio of each layer of the module for the AUC dataset. Accuracy decreases slowly with the cropping rate from 0 to 0.9, which means that the corresponding convolutional layer is relatively insensitive, and the contribution to network accuracy is relatively low. It shows the relationship between the accuracy and the pruning ratio for the AUC dataset. The inverted residual modules of con4_3, con5_2, con5_3, and con5_4 have a relatively small impact on accuracy. The modules of con7_3 and con8 have a more significant effect on accuracy. Compared with Figure 5(a), the sensitivity of MobilenetV2-tiny shown in Figure 5(b) reduces the residual bottleneck layer, whose impact on accuracy is less than 1%. We can reduce the model sublayers that have a relatively small impact on accuracy. At the same time, for precision-sensitive layers, we can increase the number of cycles of the module sublayer to improve precision.

The experimental results of the improved MobileNetV2-tiny model for the AUC dataset are shown in Table 2. Compared with the original models, the MobileNetV2-tiny model has fewer parameters. The MobileNetV2-tiny model has a 1.63% higher accuracy than the original MobileNetV2, with only 78.06% of the original MobileNetV2 parameters. This new design reduces computational complexity while maintaining the accuracy of driver posture classification, which is necessary for embedded applications. FLOPS are directly reduced by 71.6%, which is very suitable for deployment on resource-limited devices.

Table 3 shows the results compared with the latest methods in the literature. The number of parameters of our model is relatively small, while the accuracy of the network is relatively high. Our method is the optimization of the existing mature algorithm, which is a different way of improvement.

Table 4 shows the verification results for the StateFarm dataset. The StateFarm dataset was randomly split into a training data set : test data set = 9 : 1. A total of 100 epochs were trained. Input parameters are the same as those in the training AUC dataset. The improved MobileNetV2-tiny's accuracy rate is 0.19% higher than that of the original model. Other studies, such as Dhakate and Dash [22] randomly split the national train data set: test data set = 7 : 3. By dividing the dataset in this way, the results are shown in Table 5. Our method achieves the highest accuracy rate of 99.88%, and the amount of parameters is significantly reduced compared to InceptionV3 and Xception. Our method has achieved a good performance for StateFarm.  Table 6 shows the test results for the StateFarm dataset with the dataset split by 6 : 2 : 2. We achieved 99.78% in the MobileNetV2-tiny module, which shows the validity of our method.

#### 4.2.1. Training time

Both the block-level and channel-level stages of pruning add additional overheads. However, we introduce fine-tuning to avoid the complete forward and backward retraining process. Our method requires about three times the training time compared to regular training. Since our model is lightweight, there is reduced time needed for validation. We consider the increase in training time to be acceptable.

### 4.3. Results of Other Network Models

SqueezeNet1_1, EfficientNetB0, and MobileNetV3_large_x1_0 models were used to apply the proposed lightweight method to verify adaptability. The performance of accuracy and the number of parameters are summarized in Table 7. It can be observed that SqueezeNet, EfficientNet, and MobileNetV3 maintain good accuracy with design. Especially for MobileNetV3, the number of parameters has been reduced by 66.17% and the accuracy rate has reached 95.45%.

**Figure 6:**
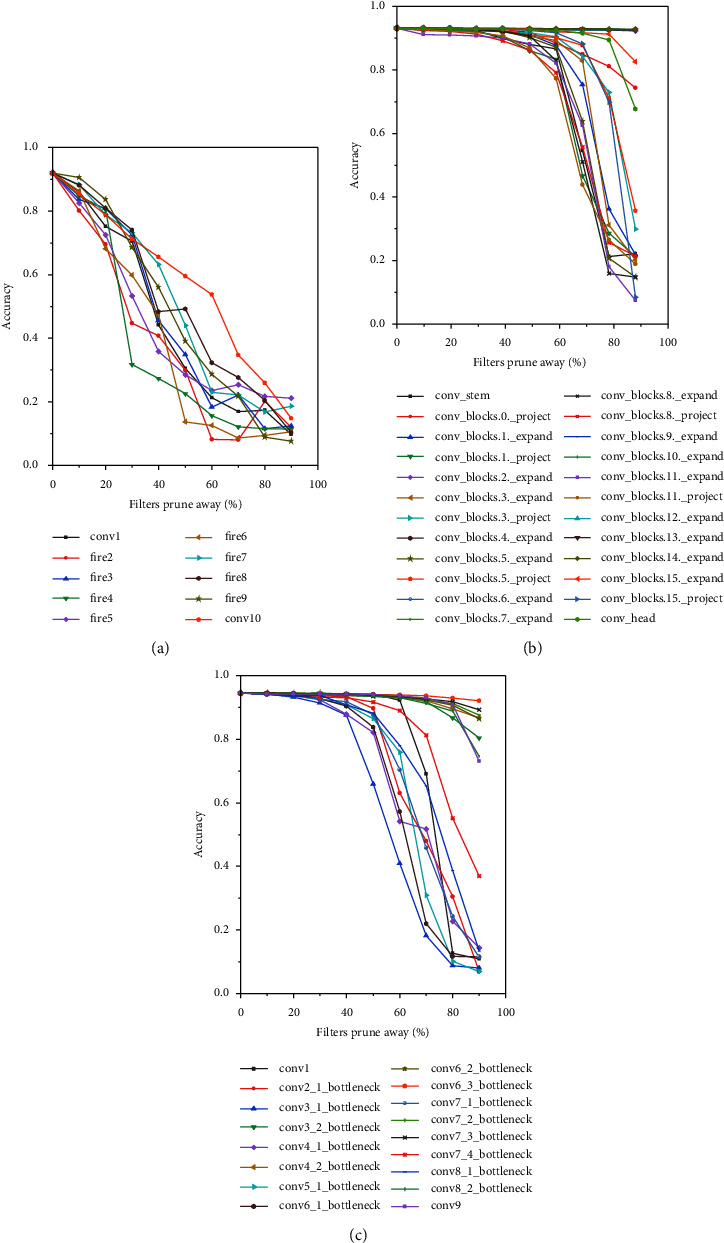
The sensitivity of SqueezeNet, EfficientNet, and MobileNetV3 for the AUC dataset. (a) The sensitivity of SqueezeNet. (b) The sensitivity of EfficientNet. (c) The sensitivity of MobileNetV3.

The accuracy of improved SqueezeNet has less improved, which may be due to SqueezeNet having fewer network layers and a reasonable and streamlined structure. It can also be seen from Figure 6 that compared with EfficientNet and MobileNetV3, each layer of SqueezeNet is susceptible to accuracy. Modifying the layers and filters of SqueezeNet affects the change in accuracy. Unlike VGG or ResNet, which are often used to demonstrate model compression, MobileNetV2, SqueezeNet, EfficientNet, and MobileNetV3 networks have relatively fewer parameters in layers. Hence, lightweighting for these networks is challenging, and even the effect is not ideal.

### 4.4. Deployment on Embedded Devices

Paddlepaddle's Paddle-Lite tool was used to assist deployment. The model was deployed on embedded devices of Xiaoyi Smart Rearview Mirror and HUAWEI MediaPad c5. Xiaoyi Smart Rearview Mirror is intelligent vehicle monitoring equipment that integrates quad-core ARM Cortex-A53 MPCoreTM with CPU MT8665 and a GPU OpenGL|ES 3.0 image processor. It is currently used in various vehicle terminals for ADAS and driving records. HUAWEI MediaPad c5 is a high-performance mobile processor with CPU HUAWEI Kirin990, a GPU Turbo 1.0 processor, and NPU dual large core + microcore computing architecture. The conversion tool Paddle-Lite converts the trained model on the server platform into an inference model that can be applied in an embedded environment, including data structure conversion and file parameter conversion.

**Figure 7:**
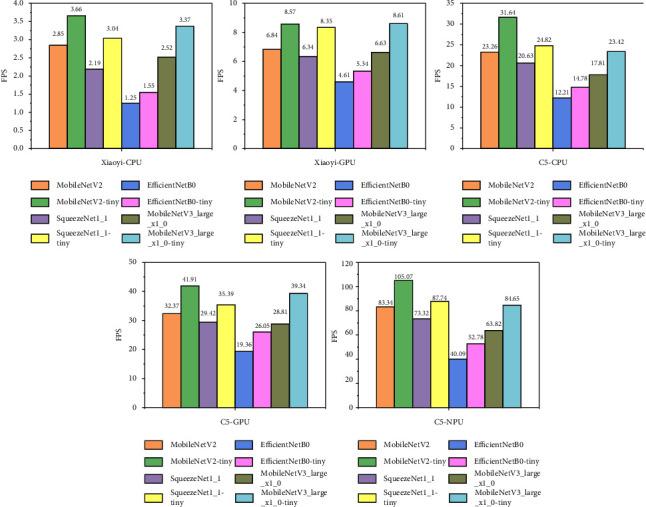
Inference FPS on resource-limited devices.

Figure 7 shows the FPS to infer frames on the embedded platform. On the Xiaoyi platform, the optimized model inference speed has been significantly improved. The processing time for one frame is faster than that of the original model, which can meet real-time processing requirements. The reasoning speed of HUAWEI MediaPad c5 is faster and can reach the level of real-time processing of video frames. In general, the proposed approach can achieve real-time processing speed and can be applied to actual distracted driving scenarios, which have a certain practical reference value for the research on the deployment of driving behaviour detection end to end.

## 5. Conclusion

Although some studies have considered distracted driving detection, the current works are less focused on real-time detection for embedded devices. We develop a lightweight design model for real-time monitoring of driving behaviour, which can be applied to vehicle-mounted terminals to provide real-time reminders. We adopted the method of pruning, but different from the direct compression of layers and filters in the past, our method was to automatically adjust the distribution of layers according to the task, which can increase the cycle of critical layers while pruning the least essential layers. The proposed MobilevetV2-tiny FLOP model is only 0.7 MobileNetV2 and obtains an accuracy of 1.63%, which is higher than that of the original MobileNetV2 model for the AUC dataset. Compared with the advanced methods in the existing literature, the results show that our method has advantages in terms of speed and model size while maintaining high accuracy. The lightweight method can meet real-time processing requirements for embedded devices.

However, there are still some problems. First, we notice that in the SqueezeNet model, the improvement of the proposed method is relatively small (only 0.51% improvement). Second, the training time of the proposed method becomes longer. (1) Next, we will continue to improve the method in order to reduce the time consumption of pruning. (2) We will further investigate other methods to improve the actual hardware efficiency of the model. In the future, we wish to comprehensively analyze the degree of dangerous driving behaviours by integrating information such as vehicle speed to meet the needs of practical applications.

## Figures and Tables

**Algorithm 1:**
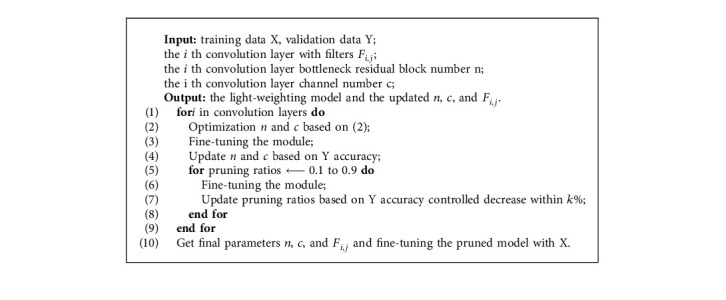
The proposed CNN light-weighting method.

**Table 1:** The architecture of MobileNetV2-tiny transforming the bottleneck residual block from n to n′, with channels ranging from c to c′.

Layer	Operator	Input	*n*	*n*′	*c*	*c*′
Conv1	Conv2d	224 × 224 × 3	1	1	32	22
Conv2	Bottleneck	112 × 112 × 32	1	1	16	11
Conv3	Bottleneck	112 × 112 × 16	2	2	24	17
Conv4	Bottleneck	56 × 56 × 24	3	2	32	22
Conv5	Bottleneck	28 × 28 × 32	4	1	64	45
Conv6	Bottleneck	14 × 14 × 64	3	3	96	67
Conv7	Bottleneck	14 × 14 × 96	3	4	160	144
Conv8	Bottleneck	7 × 7 × 160	1	2	320	288
Conv9	Conv2d	7 × 7 × 320	1	1	1280	1152
AvgPool	AvgPool	7 × 7 × 1280	1	1	—	—
Fc	Linear	1280 10	—	—	—	—

**Table 2:** The results of the improved MobileNetV2-tiny model for the AUC dataset.

Model	Source	Top-1 acc (%)	Params (M)	FLOPS (G)
MobileNetV2	AUC	93.14	3.572	0.6
MobileNetV2-tiny	AUC	**94.77**	**2.788**	**0.43**

**Table 3:** Comparisons with the state-of-the-art methods in the literature for the AUC dataset.

Model	Source	Top-1 acc (%)	Params (M)
AlexNet [20]	Original AUC	93.65	62
	Skin segmented	93.60	62
	Face	84.28	62
	Hands	89.52	62
	Face + hands	86.68	62
InceptionV3 [20]	Original AUC	95.17	24
	Skin segmented	94.57	24
	Face	88.82	24
	Hands	88.82	24
	Face + hands	90.88	24
GA weighted ensemble of all 5 [20]		95.98	120
VGG [32]	Original AUC	94.44	140
VGG with regularization [32]	Original AUC	96.31	15
Our method	Original AUC	**94.77**	**2.78**

**Table 4:** The results of the improved MobileNetV2-tiny model for the StateFarm dataset with the dataset randomly split by 9 : 1.

Model	Source	Top-1 acc(%)
MobileNetV2	StateFarm	99.62
CDCNN [6]	StateFarm	99.73
MobileNetV2-tiny	StateFarm	**99.81**

**Table 5:** Comparisons with the state-of- the-art methods in the literature for the StateFarm dataset with the dataset randomly split by 7 : 3.

Model	Top-1 acc (%)	Params (M)
InceptionV3 [22]	92.90	25.6
Xception [22]	82.50	22.9
InceptionV3 + Xception [22]	90.00	46.7
InceptionV3 + Xception + ResNet50 + VGG-19 [22]	97.00	214.3
D-HCNN [31]	99.82	0.76
MobileNetV2	99.57	3.5
MobileNetV2-tiny	**99.88**	**2.78**

**Table 6:** The results for the StateFarm test set with the dataset randomly split by 6 : 2 : 2.

Model	Source	Top-1 Acc (%)
MobileNetV2	StateFarm	99.56
MobileNetV2-tiny	StateFarm	**99.78**

**Table 7:** Performance of our method with different models for the AUC dataset.

Model	Top-1 acc (%)	Params (M)	FLOPS (G)
SqueezeNet1_1	92.03	2.26	0.69
EfficientNetB0	93.05	5.1	0.72
MobileNetV3_large_x1_0	95.15	5.47	0.45
SqueezeNet1_1-tiny	**92.54**	**2.18**	**0.64**
EfficientNetB0-tiny	**94.49**	**4.35**	**0.62**
MobileNetV3_large_x1_0-tiny	**96.01**	**4.42**	**0.33**

## Data Availability

The AUC public dataset and the StateFarm open training dataset were used for this study.
